# Human expertise or artificial intelligence? A prospective study on nail disorder diagnosis

**DOI:** 10.1038/s41746-026-02850-9

**Published:** 2026-06-02

**Authors:** Flurin L. Brand, Ali Mokhtari, Simone Cazzaniga, Christoph Brand, Cindy Franklin, Stefan Furrer, Chander Grover, Eckart Haneke, Kristine Heidemeyer, Matilde Iorizzo, Alexandra Junge, Christine A. Langhorst, Shari R. Lipner, Luigi Naldi, Talisa Luana Ragonsesi, Sanjive Rey, Bertrand Richert, Basil Signer, Charlotte Vogel, Nikhil Yawalkar, Sven Zürcher, Dominik Obrist, S. Morteza Seyed Jafari

**Affiliations:** 1 Department of Dermatology, Inselspital, Bern University Hospital, Bern, Switzerland; 2 ARTORG Center for Biomedical Engineering Research, University of Bern, Bern, Switzerland; 3 Department of Dermatology, University Teaching and Research Hospital of the University of Lucerne, Lucerne, Switzerland; 4 Department of Dermatology and Venereology, University of Cologne, Faculty of Medicine and University Hospital Cologne, Cologne, Germany; 5 Department of Dermatology and STD, University College of Medical Sciences and GTB Hospital, Delhi, India; 6 Dermaticum Freiburg, Freiburg, Germany; 7 Centro Dermatol Epidermis, Inst CUF, Matosinhos, Portugal; 8 Private Dermatology Practice, Bellinzona/Lugano, Switzerland; 9 Department of Dermatology, Weill Cornell Medicine, New York, NY USA; 10 Ospedale San Raffaele, Milan, Italy; 11 Dermatology Department – CHU Brugmann, Brussels, Belgium

**Keywords:** Computational biology and bioinformatics, Diseases, Health care, Medical research

## Abstract

Artificial intelligence (AI) shows promise in analyzing patterns of nail disease. This prospective, comparative study compared the diagnostic performance of dermatologists with that of large language models (LLMs). We evaluated the diagnostic accuracy of dermatologists and four freely available multimodal LLMs (GPT-4o, Grok 3, Claude Sonnet 4, and Gemini 2.5 Flash) using clinical images of nail diseases. Seventeen dermatologists correctly diagnosed the primary suspected diagnosis (SD) in 70.6% (95% CI: 65.5–75.2) of cases, and in 80.3% (95% CI: 75.7–84.2) of cases when considering both the SD and the differential diagnosis (SD + DD). Accuracy increased across dermatologist groups, ranging from residents (68.3% for SD + DD) to nail disease experts (96.0%). In comparison, AI models were correct in 25.0% (95% CI: 16.8–35.5) and 35.0% (95% CI: 25.5–45.9) of cases, respectively (*p* < 0.001). The AI algorithms correctly classified 13.9% of tumors and 52.3% of non-tumors (SD + DD, *p* < 0.001). Current freely available general-purpose AI models demonstrated limited reliability for standalone nail disease diagnosis in this exploratory setting and should not be used without clinical supervision. While these systems may assist in suggesting differential diagnoses, their performance remains variable and requires further validation in larger, clinically representative datasets.

## Introduction

Recent advances in artificial intelligence (AI) show promise in enhancing disease diagnosis, prognosis, treatment planning, and patient monitoring to tackle complex healthcare challenges and improve patient outcomes^1^. AI holds great promise in diagnosing nail disorders, as its capacity to analyze complex patterns and subtle morphological variations makes it particularly well-suited to the field of onychology^1^. AI excels in pattern recognition and repetitive tasks with high efficiency, significantly reducing analysis time. Nail disorders demonstrate minimal variation across racial groups; hence, AI analysis can ensure broad applicability, enhance accessibility through remote examination and imaging, while maintaining patient nail image confidentiality, and enable effective screening of histopathology specimens^1^. However, it should be noted that nail changes are three-dimensional structures, which makes consistent image acquisition and visual interpretation difficult.

Large language models (LLMs) are AI systems mainly trained on text, sometimes including images, and have shown promise in use in various areas of medicine, such as dermatology^2^. Although LLMs originally focused on natural language processing, they have now advanced to the point where they can process and generate text in a manner similar to humans^2,3^. However, their clinical reliability is limited, as LLMs may produce inaccurate, fabricated information (hallucinations), or non-contextual medical information, and cannot consistently ensure safe or evidence-based clinical recommendations, and they are not yet validated for independent diagnostic use. Unlike conventional machine learning or deep learning models, which primarily focus on image analysis, LLMs can also analyze text inputs to support clinical decision-making and improve patient-physician communication by explaining diagnostic reasoning or treatment options in plain language^2,4^. This versatility makes LLMs an exciting addition to dermatology, particularly in areas where image-based AI tools are ineffective, such as addressing patient-specific concerns or contextualizing image findings with patient histories^2^.

The availability and development of free AI systems in dermatology is rapidly growing, offering valuable support in diagnostic processes and clinical decision-making. The aim of this study was to conduct a prospective assessment of four freely available AI models, which were not specifically trained on nail diseases but show potential for detecting such conditions, and to compare their diagnostic accuracy with that of dermatology residents, board-certified dermatologists, and nail experts. The study was designed to assess the diagnostic accuracy of LLMs for nail disorders. While the findings may provide preliminary insights into the potential supportive role of AI in dermatological practice, further validation is required before drawing conclusions regarding its impact on diagnostic efficiency and accuracy.

## Results

### Participant characteristics

The characteristics of the dermatologists who participated in the study are summarized in Table 1.

**Table 1:** Characteristics of dermatologists included in the study

	*N* = 17 (%)
Group	
Residents	6 (35.3)
Board-certified dermatologists	6 (35.3)
Experts in nail diseases	5 (29.4)
Experience	
1–10 years	7 (41.2)
11–20 years	3 (17.6)
21+ years	7 (41.2)
Working place	
University hospital	11 (64.7)
Public hospital	4 (23.5)
Private hospital	1 (5.9)
Private practice	1 (5.9)

### Overall diagnostic accuracy

Overall, the dermatologists answered correctly in 70.6% (95% CI: 65.5, 75.2) and 80.3% (75.7, 84.2) of the cases for SD and SD + DD, respectively, while AI answered correctly in 25.0% (16.8, 35.5) and 35.0% (25.5, 45.9) of the cases, respectively (Table 2). These differences were statistically significant (*p* < 0.001, Supplementary Table 1).

**Table 2:** Correct answers for each group of evaluators, in total and in detail

Group	SD	SD + DD
	% (95% CI)	% (95% CI)
Dermatologists	**70.6 (65.5, 75.2)**	**80.3 (75.7, 84.2)**
Residents	58.3 (49.4, 66.8)	68.3 (59.6, 76.0)
Board-certified dermatologists	70.8 (62.2, 78.2)	79.2 (71.1, 85.5)
Experts in nail diseases	85.0 (76.7, 90.7)	96.0 (90.2, 98.4)
AI	**25.0 (16.8, 35.5)**	**35.0 (25.5, 45.9)**
ChatGPT	25.0 (11.2, 46.9)	30.0 (14.5, 51.9)
Grok	15.0 (5.2, 36.0)	25.0 (11.2, 46.9)
Gemini	35.0 (18.1, 56.7)	50.0 (29.9, 70.1)
Claude	25.0 (11.2, 46.9)	35.0 (18.1, 56.7)

The bold values represent the aggregated results of the two main evaluator groups (all dermatologists combined and all AI models combined).

*AI* artificial intelligence algorithms, *CI* confidence interval, *DD* differential diagnosis, *SD* suspected diagnosis.

### Performance by evaluator group

**Fig. 1:**
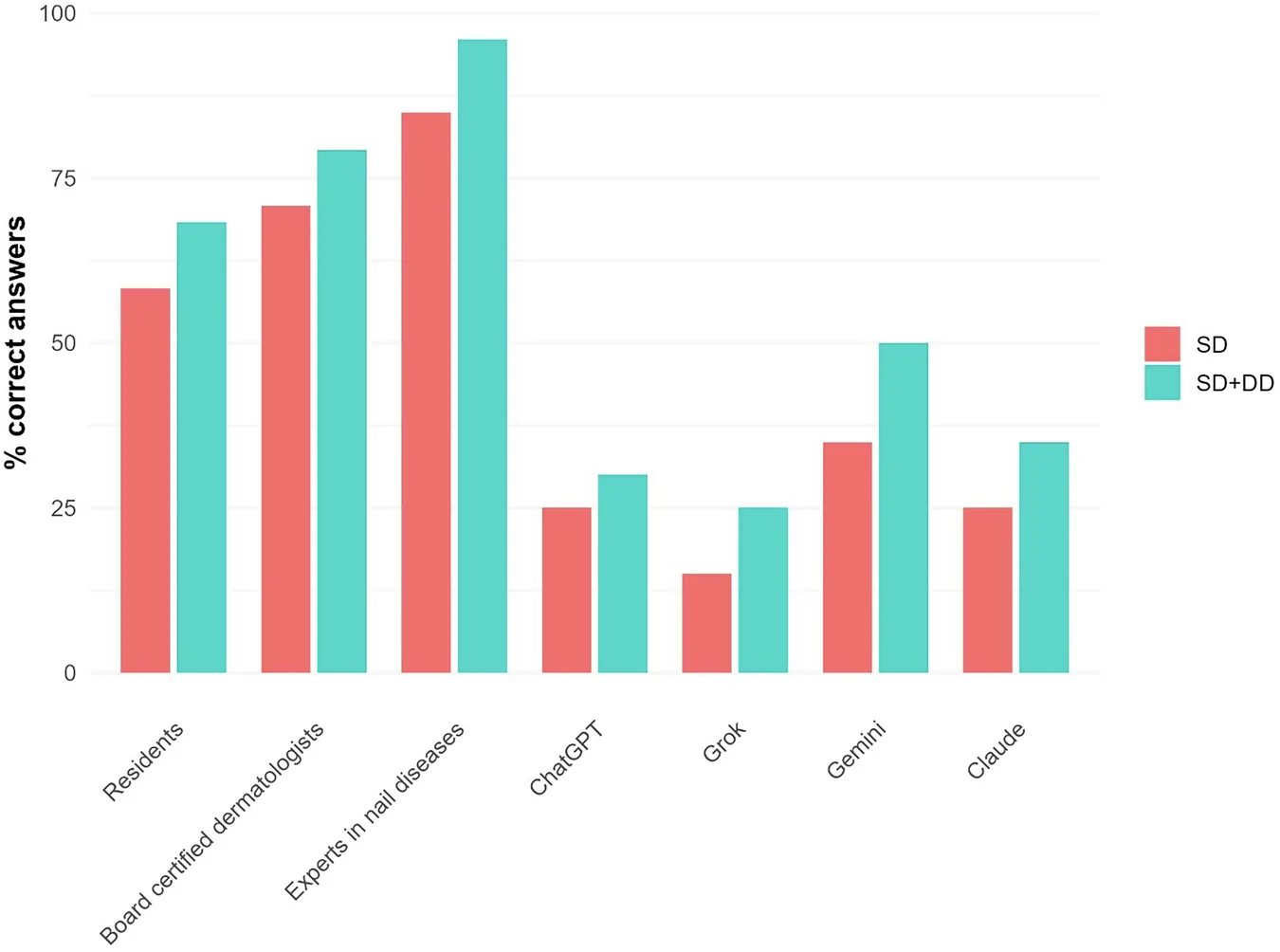
Correct answers for each group of evaluators in detail.

Considering the accuracy of each group of dermatologists, there was an increasing trend from residents (68.3% for SD + DD) to experts in nail diseases (96.0%) (Fig. 1), with significant differences between residents and experts and between board-certified dermatologists and experts. Nonetheless, these comparisons may partly reflect small-sample variation due to the limited number of participants (*n* = 17). Regarding AI, the performance of the algorithms was comparable, with no significant differences among them. However, Gemini performed slightly better (50.0% for SD + DD). Detailed case-level results for each evaluator group are provided in Supplementary Table 2, allowing a more granular assessment of performance across individual cases.

Interestingly, the residents demonstrated a high level of diagnostic accuracy for conditions commonly encountered in dermatology clinics, such as onychomycosis, psoriasis, subungual hematomas, and melanocytic lesions like longitudinal melanonychia and melanoma. However, they struggled considerably more with correctly identifying rare conditions, including onychomatricoma, fibromyxoma, subungual exostosis, and median canaliform nail dystrophy (onycholysis mediana canaliformis). In contrast, board-certified dermatologists and nail specialists exhibited significantly fewer difficulties in diagnosing these uncommon disorders.

### Tumor versus non-tumor performance

When comparing tumor (*n* = 9) versus non-tumor nail lesions (*n* = 11), AI algorithms classified correctly 13.9% (6.1, 28.7) of the tumors versus 52.3% (37.9, 66.2) of the non-tumors (SD + DD, *p* < 0.001). In contrast, dermatologists classified correctly 74.5% (67.1, 80.8) of the tumors versus 85.0% (79.2, 89.4) of the non-tumors (*p* = 0.02). Among neoplastic lesions, when comparing malignant (*n* = 2) versus benign tumors (*n* = 7), AI accurately categorized 50.0% (21.5, 78.5) of the malignant versus 3.6% (0.6, 17.7) of the benign lesions (SD + DD, *p* = 0.005), whereas dermatologists classified correctly 88.2% (73.4, 95.3) of the malignant versus 70.6% (61.9, 78.0) of the benign tumors (SD + DD, *p* = 0.04). Due to the extremely small number of malignant cases, these results should be interpreted with caution. No significant difference for SD + DD was detected in both AI and dermatologists when analyzing inflammatory conditions (*n* = 3) versus infections (*n* = 2) (Supplementary Table 3).

### Inter-rater agreement

The inter-rater agreement on correct answers, in total and by group of evaluators, is described in Table 3. According to the AC1 statistic, the overall agreement among dermatologists for SD and SD + DD was 0.51 (0.27, 0.75) and 0.66 (0.45, 0.87), respectively. Agreement tended to increase with the level of experience, ranging from 0.57 for residents (SD + DD) to 0.92 for experts, without particular heterogeneity between groups. AI algorithms also showed a good level of mutual agreement, with AC1 = 0.63 (0.34, 0.92) for SD and 0.66 (0.39, 0.94) for SD + DD. However, the agreement between dermatologists and AI was poor (AC1 < 0) for both SD and SD + DD.

**Table 3:** Inter-rater agreement on correct answers, in total and by group of evaluators

Group	SD	SD + DD
	AC1 (95% CI)	AC1 (95% CI)
Dermatologists	0.51 (0.27, 0.75)	0.66 (0.45, 0.87)
Residents	0.46 (0.20, 0.72)	0.57 (0.29, 0.84)
Board-certified dermatologists	0.64 (0.39, 0.90)	0.74 (0.53, 0.94)
Experts in nail diseases	0.80 (0.62, 0.98)	0.92 (0.83, 1.00)
*Between groups*	*0.54 (0.21, 0.87)*	*0.69 (0.42, 0.95)*
AI	0.63 (0.34, 0.92)	0.66 (0.39, 0.94)
Dermatologists versus AI	−0.08 (−0.58, 0.43)	−0.27 (−0.77, 0.22)

*AI* artificial intelligence algorithms, *DD* differential diagnosis, *SD* suspected diagnosis.

## Discussion

By analyzing large datasets of easily acquired nail images and clinical data, AI systems have the potential to detect subtle abnormalities with high accuracy and uncover novel correlations with health conditions, enabling early detection and personalized treatment strategies^1^. However, our findings do not support the claim that current multimodal LLM-based systems can reliably detect subtle clinical abnormalities. Transfer learning-based computer-aided diagnostic systems have proven effective in general identification of various multi-lesion nail diseases, such as koilonychia, Beau’s lines, leukonychia, yellow nail syndrome, and psoriasis, with varying degrees of accuracy^1,5–7^. Specifically, deep learning-based diagnostic models have also been developed for onychomycosis, a condition that is well-suited to AI-based diagnosis due to its high prevalence across populations^8–11^. In addition to fungal nail infections, AI applications have also shown promising results in the assessment of nail psoriasis. Folle et al. developed a deep learning model to automatically quantify the modified nail psoriasis severity index and achieved high agreement with human experts^12^. It is important to clearly distinguish between AI models developed specifically for image analysis and general-purpose LLMs, which are primarily trained for text-based reasoning and not optimized for medical image interpretation. LLMs have attracted growing attention for their potential to improve the efficiency and quality of clinical diagnosis and therapeutic management decisions^13^. Although LLMs have shown promise in aiding clinicians with the diagnosis of skin diseases, their accuracy in diagnosing nail diseases remains exploratory and unvalidated. To address this issue, we conducted an exploratory study to evaluate the ability of common general-purpose LLMs to diagnose nail diseases.

The results of the current study demonstrate that physicians, particularly those with specialized experience in nail disorders, achieve substantially higher diagnostic accuracy than general-purpose AI models when interpreting nail disease images. The accuracy increased in proportion to clinical expertise, with nail specialists achieving nearly perfect diagnostic rates, especially when differential diagnoses were considered. However, these results must be interpreted with caution, as they were based on a small dataset (20 cases) and a limited number of experts, which limits the generalizability of the findings. Therefore, this study should be considered exploratory and hypothesis-generating rather than definitive. Once a correct diagnosis was established, physicians consistently recommended guideline-concordant diagnostic and therapeutic steps. These results suggest that clinical expertise not only aids in accurate identification but also ensures appropriate patient management. In contrast, the AI models underperformed markedly. While they occasionally generated plausible diagnoses and treatment suggestions when correct, their accuracy, especially for nail tumors, was limited.

Interestingly, Gemini 2.5 Flash showed a better performance than other AI models, suggesting that variations in architecture, image processing, or training corpus may influence model performance, even among general-purpose systems. However, it should be acknowledged that the image preprocessing pipeline may have disproportionately affected AI performance compared to human observers, potentially introducing a systematic bias in favor of human interpretation. Furthermore, it seems that all of the AI tools tested lack the specialized training necessary for consistent and reliable performance in diagnosing nail diseases. Nevertheless, the exact datasets, training models, and proportions of the different LLMs remain undisclosed. These results demonstrate that currently available general-purpose AI models, when used to analyze patient photographs without additional clinical context, do not yet match the diagnostic performance of trained medical professionals in identifying nail diseases. Especially in the evaluation of rare or inflammatory nail conditions, expert human judgment remains essential. This observation aligns with existing literature indicating that AI models tend to perform better with well-represented, visually distinct conditions and struggle with rare or inflammatory diseases due to limited training data and greater clinical heterogeneity^8,11,14^. These results are also consistent with prior studies showing that, although deep learning models can match or exceed dermatologists’ accuracy in controlled settings^8,11^, their performance in real-world clinical tasks remains variable, and their generalizability decreases significantly on external datasets, particularly for underrepresented skin types and less common conditions^15^.

Our findings suggest that currently available general-purpose multimodal LLMs show limited performance for nail disease image interpretation under the conditions of this exploratory study. This limited performance may be related to insufficient representation of high-quality, diverse, and clinically curated data in training, particularly with regard to different skin tones and rare conditions. Their performance is further limited by poor generalization to real-world, variable-quality images and by their inability to incorporate essential clinical context, such as clinical history, patients’ symptoms, and lesion evolution. While AI models often generate appropriate treatment recommendations, especially when the suspected diagnosis is correct, they often exhibit overconfidence and may produce incorrect or misleading outputs, posing risks in clinical settings. This is especially important when a nail tumor is misdiagnosed as onychomycosis. This critical issue is highlighted in the present study, as the AI algorithms could correctly classify 13.9% of the tumors compared to 52.3% of the non-tumors (SD + DD, *p* < 0.001). For example, several models have suggested initiating topical or systemic antifungal therapy for cases diagnosed as subungual melanoma or other nail tumors. In practice, this can lead to delayed presentation to a dermatologist and an increased risk of tumor progression. This underlines the importance of control and integration of AI information into clinical workflows by medical staff. For the future, this indicates the need to develop robust safeguards to prevent the uncritical adoption of AI-generated recommendations into patient care.

Notably, the inter-rater agreement between nail experts reached very high values (AC1 up to 0.92), underlining the exceptional reproducibility and reliability of visual assessment by specialists with specialized experience in onychology. This high level of agreement suggests that when confronted with standardized photographic material, the experts recognize and interpret relevant morphological features in a very similar way. However, this agreement was derived from a small number of standardized images and may not reflect real-world variability. In contrast, the low level of agreement between AI systems and physicians, as reflected by AC1 scores consistently below zero, highlights a fundamental discrepancy in diagnostic reasoning between algorithmic and clinical assessments. This divergence is likely due to differences in importance attribution, whereby AI models may overemphasize image elements that are clinically irrelevant or misinterpret artifacts as diagnostic clues. Interestingly, the AI systems themselves showed moderate inter-rater agreement (AC1 approximately 0.66), suggesting that they apply relatively consistent internal decision rules for all cases, despite their limited overall diagnostic accuracy. This observation may indicate that common model architectures, shared training data corpora, or similar training goals may predispose AI systems to systematic—albeit inaccurate—diagnostic patterns for general purposes. These tendencies could further limit their clinical utility unless they are retrained or fine-tuned with domain-specific datasets.

Another significant issue with current LLMs in dermatology is the ethical and regulatory challenges surrounding their use^16,17^. Entrusting AI systems with sensitive protected health information raises serious concerns about privacy violations, potential misuse of personal data, and cybersecurity threats^16^. Furthermore, errors made by these models create complex questions about responsibility and liability, and could potentially endanger patient safety^16^. Lastly, the opaque use of these technologies may compromise patient autonomy and undermine patient-physician confidence^16,18^.

This study is not without limitations, the main one being the use of publicly available images, which may be known to or detectable by LLMs. However, we attempted to address this limitation by developing a structured, reproducible image preprocessing pipeline to create a diagnostically valid dataset that is distinct from any public sources. This minimized the risk that the AI model’s performance was evaluated based on its ability to generalize to new data, rather than memorizing previously seen images. This preprocessing pipeline, although designed to reduce the risk of data leakage from publicly available images and to simulate real-world variability, may have affected AI systems differently than human observers, although both groups (the AI and the human participants) were presented with the same images. While diagnostically relevant features were preserved and confirmed by independent dermatologists, such transformations could introduce a systematic bias by disproportionately degrading AI performance compared to human observers. Importantly, this experimental setting does not fully reflect real-world conditions, where AI systems typically operate on unprocessed clinical images. This potential limitation should be considered when interpreting the results. Future studies with larger sample sizes, more participants, and additional clinical images may enhance the robustness and applicability of our results. The study design also reflects an unresolved ethical dilemma regarding the use of patient images in LLM platforms—an important issue that requires further ethical and technical discussion in the future.

Another limitation of this study is the absence of dermoscopic images. Dermoscopy is known to improve diagnostic accuracy in nail disorders, particularly for pigmented and tumorous lesions. However, we deliberately used standard clinical photographs to reflect real-world conditions, as these are more commonly available in routine practice and patient-generated images. Importantly, the absence of dermoscopic images applied equally to both AI models and human participants, ensuring that neither group had an inherent advantage in this regard. Future studies should incorporate dermoscopic imaging to further enhance AI-based diagnostic performance.

Future research should focus on the development of domain-specific AI models trained on curated datasets of nail diseases to improve diagnostic accuracy and generalizability. The integration of multimodal data, including clinical history, dermoscopic imaging, and longitudinal follow-up, may further enhance model performance and clinical relevance. Furthermore, collaborative human-AI approaches—rather than autonomous systems—are likely to represent the most effective strategy for safe and meaningful implementation in clinical practice. Standardized, high-quality image acquisition and validation on diverse, real-world datasets will also be essential to ensure robustness and applicability across different patient populations.

In summary, current freely available general-purpose AI models demonstrated limited reliability for standalone nail disease diagnosis in this exploratory setting and should not be used without clinical supervision. While these systems may assist in suggesting differential diagnoses or guiding non-specialist users toward plausible considerations, their performance remains variable and requires further validation in larger, clinically representative datasets. Although LLMs show strong diagnostic performance for some nail diseases, they still struggle with rare conditions, posing a persistent challenge for current models. Future developments of LLMs should therefore focus on training domain-specific models for nail diagnostics, with integration of multimodal clinical data to improve performance and clinical relevance. Future research should include larger, clinically representative datasets to enable more robust training, validation, and assessment of AI models in onychology.

## Methods

### Study participants

This prospective, comparative study was conducted across several specialized dermatology centers worldwide. It aimed to evaluate the diagnostic accuracy of dermatology residents, board-certified dermatologists, and experts in nail diseases compared to AI models. The proposed diagnostic steps and recommended treatments were also analyzed and compared. The human participants were divided into three groups: Group-1 consisted of six dermatology residents; Group-2 included six board-certified dermatologists; and Group-3 comprised five specialists dedicated to diagnosing and treating nail diseases. Although the total number of participants was limited, the groups were selected to represent different levels of dermatological expertise. The physicians were shown 20 pictures of nail diseases and asked a series of questions. First: “What is your first suspected diagnosis?”, followed by: “Please name other differential diagnoses in order of importance (maximum 3)”, then: “What further diagnostic steps would you suggest?” and finally: “What treatment would you recommend?”

Four well-known, publicly available multimodal AI models were also used for image-based diagnosis: ChatGPT-4o (OpenAI), Claude Sonnet 4 (Anthropic), Gemini 2.5 Flash (Google DeepMind), and Grok 3 (xAI). These models were confronted with the same questions; one run was performed for each AI model. The models were evaluated for diagnostic accuracy and clinical applicability compared to human participants. It is important to note that these models are not specifically trained for medical image-based diagnosis. The primary outcome of the study was diagnostic accuracy (SD and SD + DD). The additional questions regarding diagnostic steps and treatment recommendations were considered secondary. These responses were generally consistent with the respective suspected diagnoses, but not necessarily with the correct diagnosis; therefore, no separate quantitative analysis of these components was performed, and they were considered exploratory.

### Preparation of the clinical cases

Freely available images of nail diseases were collected from public sources (i.e., case reports and textbooks) with diagnoses confirmed by direct microscopy, fungal culture, or histopathology. The dataset enables unbiased comparison between AI and experts while preventing leakage from overlapping with AI training images, a common risk when reusing public dermatology datasets.

We developed a Python-based preprocessing pipeline using OpenCV, NumPy, PIL, SciPy, and PyTorch to preserve diagnostically relevant features for human interpretation while reducing the likelihood of direct retrieval or memorization of publicly available source images. Each image was cropped by approximately 0.75% per side to remove border artifacts, and padded by approximately 1.5% with a white background. Geometric transformations, simulating natural photographic variation, included random scaling between 0.9 and 1.1 times and rotation within ±3°. Photometric adjustments slightly increased brightness and contrast (between 1.01 and 1.04 times), enhanced saturation (between 1.005 and 1.01 times), and applied gamma correction in the range of 0.8 to 1.2 to mimic variation in lighting and color environments. To further reduce recognizability, a light Gaussian blur (kernel size 3), low-variance Gaussian noise, and a low-intensity smoothed random perturbation were applied in a controlled manner. As these preprocessing steps may affect AI models differently from human evaluators, potential image degradation and systematic bias were carefully considered. This perturbation was imperceptible to humans but inspired by techniques commonly used in robustness testing of AI systems. Two independent dermatologists reviewed all altered images and confirmed that essential clinical features were preserved and that the images retained diagnostic value. However, validation by only two reviewers may not fully eliminate this issue. The final dataset maintained lesion characteristics while incorporating subtle geometric, color, and pixel-level variations.

Four publicly accessible multimodal AI systems, ChatGPT-4o (OpenAI), Claude Sonnet 4 (Anthropic), Gemini 2.5 Flash (Google DeepMind), and Grok 3 (xAI), were tested on this dataset, representing tools that healthcare providers or laypersons might realistically consult. This approach enabled a fair and controlled evaluation of AI diagnostic potential against expert clinical judgment.

### Statistical analysis

Data were presented as absolute numbers with percentages for descriptive purposes. The answers of the participants and the AI algorithms were compared against the true available information about the presented case. The correctness of the primary suspected diagnosis (SD) alone and of the combined SD and differential diagnosis (DD) (SD + DD), which evaluated at least one of the two, was assessed as a binary response (yes versus no). Only medically valid diagnoses were considered correct. Responses that listed symptoms instead of diagnoses (e.g., onycholysis instead of psoriasis) were considered incorrect. Differential diagnoses were only considered correct if they were explicitly stated and medically accurate. Cases in which no differential diagnoses were listed were classified as incorrect unless the primary suspected diagnosis was correct. Correct answers were presented as percentages of the total evaluators and cases, along with their 95% confidence intervals (CI). Comparisons of correct answers by group of evaluators or type of condition were performed by using Pearson’s Χ2 test or Fisher’s exact test in places where required. In case of statistical significance, subgroup comparisons among evaluators were also performed with Holm–Bonferroni correction. The inter-rater agreement, which measures the consistency of evaluators in answering correctly or incorrectly on the same cases, was assessed using Gwet’s AC1 statistic with 95%^19^. The interpretation of AC1 is similar to kappa and can be read as follows: <0 poor, 0–0.20 slight, 0.21–0.40 fair, 0.41–0.60 moderate, 0.61–0.80 good, 0.81–1 very good agreement. Statistical analysis was conducted with R software version 4.3.3 (R Foundation for Statistical Computing, Vienna, Austria).

### Ethics and consent

This study evaluated diagnostic responses of dermatologists and AI systems using clinical images of nail diseases obtained from publicly available sources. According to local institutional requirements, formal ethics approval was waived because no identifiable patient data were used and no intervention involving patients was performed. The use of publicly available, non-identifiable clinical images did not require informed consent.

## Supplementary information

Supplementary Material

## Data Availability

The data supporting the findings of this study are provided within the article and its Supplementary Information.

## Code availability

The code used for image preprocessing and analysis is available from the corresponding author upon reasonable request.
