# Supplementary material for: Human expertise or artificial intelligence? A prospective study on nail disorder diagnosis
Source: NPJ Digit Med. 2026 Jun 2;9:705. doi: 10.1038/s41746-026-02850-9 (PMC13578321; doi:10.1038/s41746-026-02850-9)
Supplement: Supplementary file 1 [file 41746_2026_2850_MOESM1_ESM.pdf]

Supplementary Material

Study Questionnaire

The physicians and AI interviewed were shown 20 different pictures of nail diseases and then asked a series of questions:

1. What is your first suspected diagnosis?
2. Please name other differential diagnoses in order of importance (maximum 3)?
3. What further diagnostic steps would you suggest
4. What treatment would you recommend?

Supplementary Material

Study Cases

The following diagnoses were included in the patient images shown:

1. Congential malalignment of the big toenail
2. Acral fibrokeratoma
3. Green nail syndrome (pseudomonas infection)
4. Lichen planus unguium
5. Myxoid pseudocyst
6. Longitudinal melanonychia due to melanocyte activation
7. Nail psoriasis
8. Onychomatricoma
9. Nail psoriasis
10. Onychomycosis
11. Periungueal viral wart
12. Onychopapilloma
13. Nail overcurvature (pincer nail)
14. Acral fibromyxoma
15. Periungueal viral wart
16. Subungual hematoma
17. Subungual exostosis
18. Melanoma
19. Squamous cell carcinoma of the nail unit
20. Onychodystrophia mediana canaliformis

Supplementary Tables

Supplementary Table 1 - Comparisons of correct answers by group of evaluators

Group	SD	SD+DD
	p*	p*
Dermatologists (among all subgroups)	<0.001	<0.001
Residents vs Board certified dermatologists	0.06	0.08
Residents vs Experts in nail diseases	<0.001	<0.001
Board certified dermatologists vs Experts in nail diseases	0.04	0.01
Dermatologists vs AI	<0.001	<0.001
AI (among all algorithms)	0.55	0.38

AI: artificial intelligence algorithms, DD: differential diagnosis, SD: suspected diagnosis

* Pearson's X^2 test or Fisher's exact test if required. For subgroup comparisons p-values were adjusted by Holm–Bonferroni method.

Supplementary Table 2 - Correct answers for each case, in total and by group of evaluators

Case	Total Dermatologists (N = 17)		Dermatology residents (N = 6)		Board certified dermatologists (N = 6)		Experts in nail diseases (N = 5)		AI (N = 4)	
	SD	SD+DD	SD	SD+DD	SD	SD+DD	SD	SD+DD	SD	SD+DD
1	47.1 (26.2, 69.0)	58.8 (36.0, 78.4)	16.7 (3.0, 56.4)	33.3 (9.7, 70.0)	33.3 (9.7, 70.0)	50.0 (18.8, 81.2)	100 (56.6, 100)	100 (56.6, 100)	0.0 (0.0, 49.0)	0.0 (0.0, 49.0)
2	82.4 (59.0, 93.8)	82.4 (59.0, 93.8)	50.0 (18.8, 81.2)	50.0 (18.8, 81.2)	100 (61.0, 100)	100 (61.0, 100)	100 (56.6, 100)	100 (56.6, 100)	0.0 (0.0, 49.0)	0.0 (0.0, 49.0)
3	100 (81.6, 100)	100 (81.6, 100)	100 (61.0, 100)	100 (61.0, 100)	100 (61.0, 100)	100 (61.0, 100)	100 (56.6, 100)	100 (56.6, 100)	50.0 (15.0, 85.0)	50.0 (15.0, 85.0)
4	94.1 (73.0, 99.0)	94.1 (73.0, 99.0)	100 (61.0, 100)	100 (61.0, 100)	83.3 (43.6, 97.0)	83.3 (43.6, 97.0)	100 (56.6, 100)	100 (56.6, 100)	0.0 (0.0, 49.0)	50.0 (15.0, 85.0)
5	82.4 (59.0, 93.8)	82.4 (59.0, 93.8)	50.0 (18.8, 81.2)	50.0 (18.8, 81.2)	100 (61.0, 100)	100 (61.0, 100)	100 (56.6, 100)	100 (56.6, 100)	0.0 (0.0, 49.0)	0.0 (0.0, 49.0)
6	100 (81.6, 100)	100 (81.6, 100)	100 (61.0, 100)	100 (61.0, 100)	100 (61.0, 100)	100 (61.0, 100)	100 (56.6, 100)	100 (56.6, 100)	75.0 (30.1, 95.4)	75.0 (30.1, 95.4)
7	88.2 (65.7, 96.7)	100 (81.6, 100)	100 (61.0, 100)	100 (61.0, 100)	66.7 (30.0, 90.3)	100 (61.0, 100)	100 (56.6, 100)	100 (56.6, 100)	50.0 (15.0, 85.0)	100 (51.0, 100)
8	29.4 (13.3, 53.1)	29.4 (13.3, 53.1)	0.0 (0.0, 39.0)	0.0 (0.0, 39.0)	0.0 (0.0, 39.0)	0.0 (0.0, 39.0)	100 (56.6, 100)	100 (56.6, 100)	0.0 (0.0, 49.0)	0.0 (0.0, 49.0)
9	70.6 (46.9, 86.7)	94.1 (73.0, 99.0)	50.0 (18.8, 81.2)	83.3 (43.6, 97.0)	100 (61.0, 100)	100 (61.0, 100)	60.0 (23.1, 88.2)	100 (56.6, 100)	50.0 (15.0, 85.0)	100 (51.0, 100)
10	100 (81.6, 100)	100 (81.6, 100)	100 (61.0, 100)	100 (61.0, 100)	100 (61.0, 100)	100 (61.0, 100)	100 (56.6, 100)	100 (56.6, 100)	100 (51.0, 100)	100 (51.0, 100)
11	58.8 (36.0, 78.4)	58.8 (36.0, 78.4)	33.3 (9.7, 70.0)	33.3 (9.7, 70.0)	66.7 (30.0, 90.3)	66.7 (30.0, 90.3)	80.0 (37.6, 96.4)	80.0 (37.6, 96.4)	0.0 (0.0, 49.0)	0.0 (0.0, 49.0)
12	82.4 (59.0, 93.8)	94.1 (73.0, 99.0)	83.3 (43.6, 97.0)	83.3 (43.6, 97.0)	100 (61.0, 100)	100 (61.0, 100)	60.0 (23.1, 88.2)	100 (56.6, 100)	25.0 (4.6, 69.9)	25.0 (4.6, 69.9)
13	94.1 (73.0, 99.0)	100 (81.6, 100)	83.3 (43.6, 97.0)	100 (61.0, 100)	100 (61.0, 100)	100 (61.0, 100)	100 (56.6, 100)	100 (56.6, 100)	0.0 (0.0, 49.0)	0.0 (0.0, 49.0)
14	35.3 (17.3, 58.7)	41.2 (21.6, 64.0)	0.0 (0.0, 39.0)	0.0 (0.0, 39.0)	33.3 (9.7, 70.0)	33.3 (9.7, 70.0)	80.0 (37.6, 96.4)	100 (56.6, 100)	0.0 (0.0, 49.0)	0.0 (0.0, 49.0)
15	58.8 (36.0, 78.4)	82.4 (59.0, 93.8)	33.3 (9.7, 70.0)	66.7 (30.0, 90.3)	66.7 (30.0, 90.3)	83.3 (43.6, 97.0)	80.0 (37.6, 96.4)	100 (56.6, 100)	0.0 (0.0, 49.0)	0.0 (0.0, 49.0)
16	94.1 (73.0, 99.0)	100 (81.6, 100)	83.3 (43.6, 97.0)	100 (61.0, 100)	100 (61.0, 100)	100 (61.0, 100)	100 (56.6, 100)	100 (56.6, 100)	75.0 (30.1, 95.4)	100 (51.0, 100)
17	41.2 (21.6, 64.0)	52.9 (31.0, 73.8)	16.7 (3.0, 56.4)	50.0 (18.8, 81.2)	16.7 (3.0, 56.4)	16.7 (3.0, 56.4)	100 (56.6, 100)	100 (56.6, 100)	0.0 (0.0, 49.0)	0.0 (0.0, 49.0)
18	100 (81.6, 100)	100 (81.6, 100)	100 (61.0, 100)	100 (61.0, 100)	100 (61.0, 100)	100 (61.0, 100)	100 (56.6, 100)	100 (56.6, 100)	50.0 (15.0, 85.0)	50.0 (15.0, 85.0)
19	29.4 (13.3, 53.1)	76.5 (52.7, 90.4)	33.3 (9.7, 70.0)	83.3 (43.6, 97.0)	16.7 (3.0, 56.4)	66.7 (30.0, 90.3)	40.0 (11.8, 76.9)	80.0 (37.6, 96.4)	25.0 (4.6, 69.9)	50.0 (15.0, 85.0)
20	23.5 (9.6, 47.3)	58.8 (36.0, 78.4)	33.3 (9.7, 70.0)	33.3 (9.7, 70.0)	33.3 (9.7, 70.0)	83.3 (43.6, 97.0)	0.0 (0.0, 43.4)	60.0 (23.1, 88.2)	0.0 (0.0, 49.0)	0.0 (0.0, 49.0)
Total	*70.6 (65.5, 75.2)*	*80.3 (75.7, 84.2)*	*58.3 (49.4, 66.8)*	*68.3 (59.6, 76.0)*	*70.8 (62.2, 78.2)*	*79.2 (71.1, 85.5)*	*85.0 (76.7, 90.7)*	*96.0 (90.2, 98.4)*	*25.0 (16.8, 35.5)*	*35.0 (25.5, 45.9)*

AI: artificial intelligence algorithms, DD: differential diagnosis, SD: suspected diagnosis

Results are presented as % of correct answers with 95% confidence intervals in brackets

Supplementary Table 3 - Total correct answers for each group of evaluators, by non-tumor vs tumor lesions, benign vs malignant tumors, and infections vs inflammatory conditions

Group	SD	SD	p*	SD+DD	SD+DD	p*
	**Non-tumor (N = 11)**	**Tumor (N = 9)**		**Non-tumor (N = 11)**	**Tumor (N = 9)**	
Dermatologists	76.5 (69.9, 82.0)	63.4 (55.5, 70.6)	0.008	85.0 (79.2, 89.4)	74.5 (67.1, 80.8)	0.02
AI	36.4 (23.8, 51.1)	11.1 (4.4, 25.3)	0.009	52.3 (37.9, 66.2)	13.9 (6.1, 28.7)	<0.001
	**Benign tumor (N = 7)**	**Malignant tumor (N = 2)**		**Benign tumor (N = 7)**	**Malignant tumor (N = 2)**	
Dermatologists	63.0 (54.1, 71.2)	64.7 (47.9, 78.5)	>0.99	70.6 (61.9, 78.0)	88.2 (73.4, 95.3)	0.04
AI	3.6 (0.6, 17.7)	37.5 (13.7, 69.4)	0.03	3.6 (0.6, 17.7)	50.0 (21.5, 78.5)	0.005
	**Infection (N = 2)**	**Inflammatory (N = 3)**		**Infection (N = 2)**	**Inflammatory (N = 3)**	
Dermatologists	100.0 (89.8, 100.0)	84.3 (72.0, 91.8)	0.02	100.0 (89.8, 100.0)	96.1 (86.8, 98.9)	0.51
AI	75.0 (40.9, 92.9)	33.3 (13.8, 60.9)	0.17	75.0 (40.9, 92.9)	83.3 (55.2, 95.3)	>0.99

AI: artificial intelligence algorithms, DD: differential diagnosis, N: number of conditions examined, SD: suspected diagnosis

Results are presented as % of correct answers with 95% confidence intervals in brackets

* Pearson's X^2 test or Fisher's exact test if required
